# Melatonin ameliorates age-related sarcopenia by inhibiting fibrogenic conversion of satellite cell

**DOI:** 10.1186/s10020-024-00998-2

**Published:** 2024-11-30

**Authors:** Guo-Zheng Zhu, Kai Zhao, Hong-Zhou Li, Di-Zheng Wu, Yun-Biao Chen, Dong Han, Jia-Wen Gao, Xing-Yu Chen, Yong-Peng Yu, Zhi-Wei Huang, Chen Tu, Zhao-Ming Zhong

**Affiliations:** 1 Division of Spine Surgery, Department of Orthopaedics, Nanfang Hospital, Southern Medical University, 1838 North Guangzhou Ave, Guangzhou, 510515 People’s Republic of China; 2 Department of Orthopaedics, First Affiliated Hospital of Gannan Medical University, Ganzhou, China; 3 Department of Orthopaedics, The First Affiliated Hospital of Guangzhou Medical University, Guangzhou, China

**Keywords:** Melatonin, Aging, Sarcopenia, Muscular fibrosis, Satellite cell

## Abstract

The fibrogenic conversion of satellite cells contributes to the atrophy and fibrosis of skeletal muscle, playing a significant role in the pathogenesis of age-related sarcopenia. Melatonin, a hormone secreted by the pineal gland, exhibits anti-aging and anti-fibrotic effects in various conditions. However, the effect of melatonin on satellite cell fate and age-related sarcopenia remains under-explored. Here, we report that melatonin treatment mitigated the loss of muscle mass and strength in aged mice, replenished the satellite cell pool and curtailed muscle fibrosis. When primary SCs were cultured in vitro and subjected to aging induction via d-galactose, they exhibited a diminished myogenic potential and a conversion from myogenic to fibrogenic lineage. Notably, melatonin treatment effectively restored the myogenic potential and inhibited this lineage conversion. Furthermore, melatonin attenuated the expression of the fibrogenic cytokine, transforming growth factor-β1, and reduced the phosphorylation of its downstream targets Smad2/3 both in vivo and in vitro. In summary, our findings show melatonin's capacity to counteract muscle decline and inhibit fibrogenic conversion in aging SCs and highlight its potential therapeutic value for age-related sarcopenia.

**Graphical Abstract:**

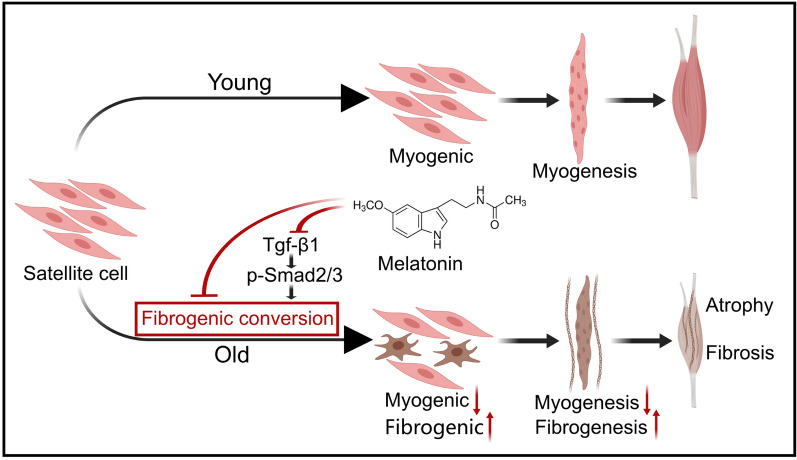

**Supplementary Information:**

The online version contains supplementary material available at 10.1186/s10020-024-00998-2.

## Introduction

Sarcopenia is a progressive skeletal muscle disorder that intensifies with age, characterized by a reduction in muscle mass and strength alongside increased muscle interstitial fibrosis (Cruz-Jentoft and Sayer 2019; Cruz-Jentoft et al. 2018). This condition significantly impacts the elderly, leading to functional decline, heightened risk of falls and fractures, frailty, and increased mortality (Malmstrom et al. 2016; Schaap et al. 2017; Bischoff-Ferrari et al. 2015). Understanding the underlying mechanisms of sarcopenia and identifying effective intervention strategies are thus of paramount importance.

Satellite cells (SCs), the resident stem cells of skeletal muscles, play a pivotal role in muscle maintenance and repair (Verdijk et al. 2014; Dumont et al. 2021; Shefer et al. 2006). Normally, SCs activate in response to muscle injury, differentiating into myoblasts to regenerate damaged tissue (Dumont et al. 2021). However, aging leads to a decline in both the number and functionality of SCs, diminishing muscle regenerative capacity and contributing to sarcopenia (Sousa-Victor and Muñoz-Cánoves 2016; Larsson et al. 2019). Moreover, aging SCs display a decline in myogenic capability and undergo a conversion from myogenic to fibrogenic lineage (Verdijk et al. 2014; Shefer et al. 2006; Almada and Wagers 2016). This fibrogenic conversion not only depletes the SC pool—causing muscle atrophy—but also increases extracellular matrix (ECM) deposition, resulting in muscle fibrosis and further impairing muscle function (Stearns-Reider et al. 2017; Zhou et al. 2019). Consequently, understanding and curbing the fibrogenic conversion of aging SCs is of prime importance.

Melatonin (MT), a methoxyindole hormone synthesized predominantly by the pineal gland, regulates circadian rhythms and exhibits anti-aging, anti-fibrotic, and antioxidant properties (Hardeland 2019; Reiter et al. 2017; Stasica et al. 1998). However, plasma melatonin levels dwindling significantly in the elderly (Reiter et al. 1980, 1981), correlating with aging and age-related diseases (Karasek and Reiter 2001). Melatonin supplementation has shown benefits in managing various conditions, enhancing quality of life (Bubenik and Konturek 2010). Specifically concerning skeletal muscles, a clinical study shows an inverse association between urine melatonin and sarcopenia in postmenopausal women, suggesting that melatonin may have a protective effect on sarcopenia (Lee et al. 2014). In previous studies, melatonin treatment facilitates muscle regeneration and recovery after injury in experimental animals (Stratos et al. 2012; Su et al. 2023). Furthermore, melatonin treatment improves muscle quality and function in both human patients and mice afflicted with Duchenne muscular dystrophy (Chahbouni et al. 2010; Hibaoui et al. 2011). However, the effects of melatonin on SC fate and age-related sarcopenia remain unclear.

In this study, we employed an aged mice model to investigate the effects of melatonin on sarcopenia. Aging SCs model, induced by d-galactose (d-gal), was also used to investigate the effects of melatonin on the fibrogenic conversion of aging SCs. This research may help to further illuminate the mechanism underlying age-related sarcopenia and provide a novel insight into the protective effects of melatonin on sarcopenia.

## Results

### Melatonin alleviated loss of muscle mass and strength in old mice

**Fig. 1:**
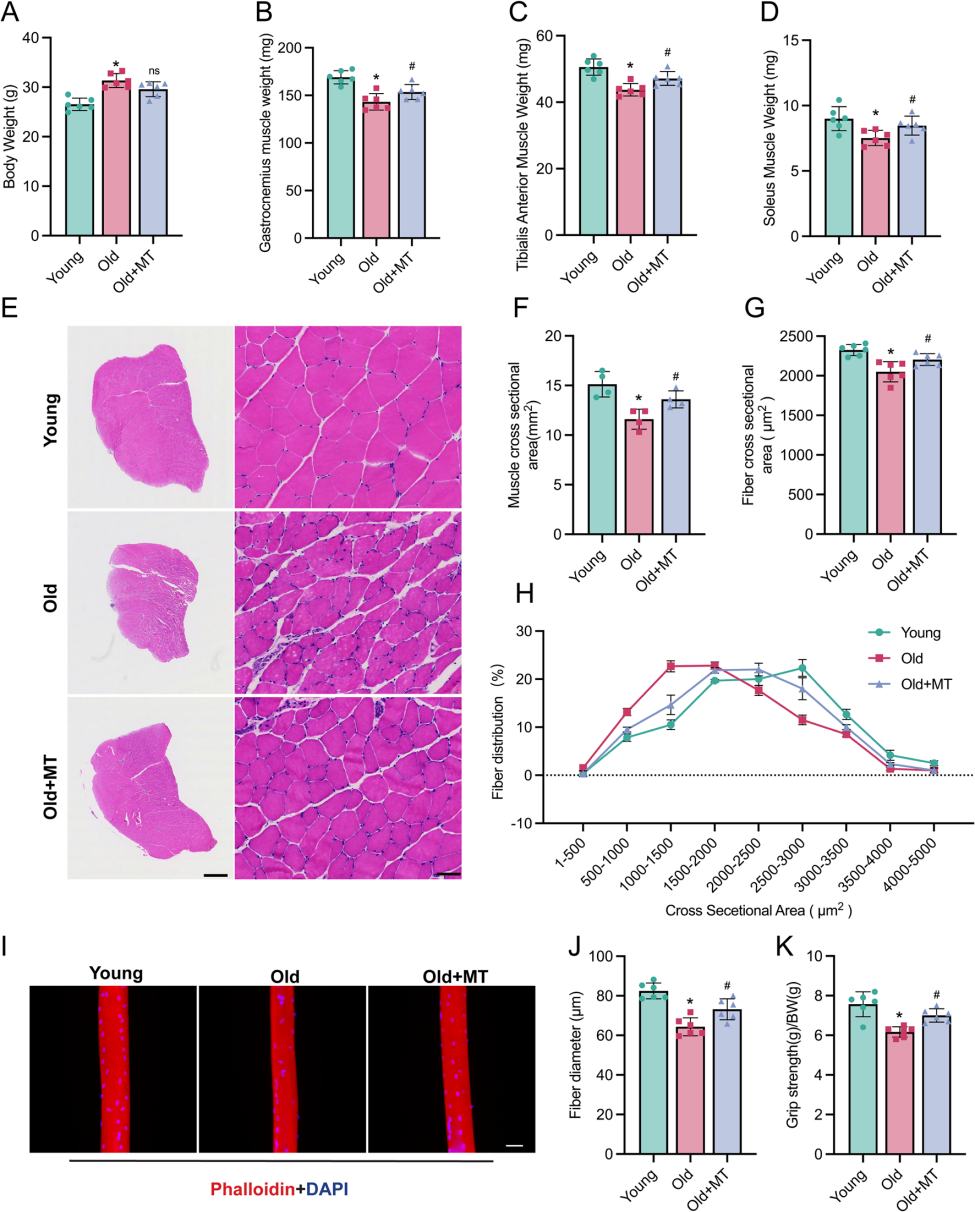
Melatonin alleviated loss of muscle mass and strength in old mice. **A** Analyses of body weight of mice. **B**–**D** Analyses of muscle weight of gastrocnemius, anterior tibialis, and soleus muscles. **E** Representative images of H&E-stained gastrocnemius muscle (Scale bar: left panel:1 mm; right panel: 50 µm). **F** Analyses of cross-sectional area of gastrocnemius muscle. **G** Analyses of average cross-sectional area of muscle fibers of gastrocnemius muscle. **H** Distribution of the cross-sectional area of muscle fibers. **I** Immunofluorescence staining of isolated muscle fibers, phalloidin (red) and nuclei (blue) (Scale bar: 50 µm). **J** Analyses of diameter of muscle fibers. **K** Analyses of forelimb grip strength of mice. Data represent mean ± S.D. of at least three independent experiments (n = 6 per/group). *p < 0.05 versus Young. ^ns^p > 0.05 versus Old. ^#^p < 0.05 versus Old

Age-related sarcopenia is characterized by decreased muscle mass and strength. To assess whether melatonin can mitigate these declines, we compared young mice (3–5 months old), aged mice (22–24 months old), and aged mice with melatonin treatment (22–24 months old, with treatment initiated at 19–21 months). Melatonin was administered daily at 11:00 AM during the light phase. Here, we found that while body weight was higher in aged mice compared to young mice, melatonin treatment did not significantly affect food intake or body weight (Fig. 1A). However, aged mice showed a reduced muscle weight of the gastrocnemius, anterior tibialis, and soleus muscles, which melatonin treatment significantly improved (Fig. 1B–D). Histological analysis revealed that aged mice had a diminished muscle cross-sectional area (CSA) and smaller average fiber CSA in the gastrocnemius muscle, both of which were increased by melatonin treatment (Fig. 1E–G). Additionally, melatonin shifted the fiber CSA distribution in aged mice closer to that of young mice (Fig. 1H). Furthermore, myofibers isolated from the aged mice had a smaller diameter than those from their younger counterparts, but melatonin effectively countered this atrophy (Fig. 1I, J). Importantly, melatonin administration significantly enhanced forelimb grip strength in aged mice, approaching levels observed in young mice (Fig. 1K). These results suggest that melatonin alleviates loss of muscle mass and strength in old mice.

### Melatonin restored satellite cell pool in old mice

**Fig. 2:**
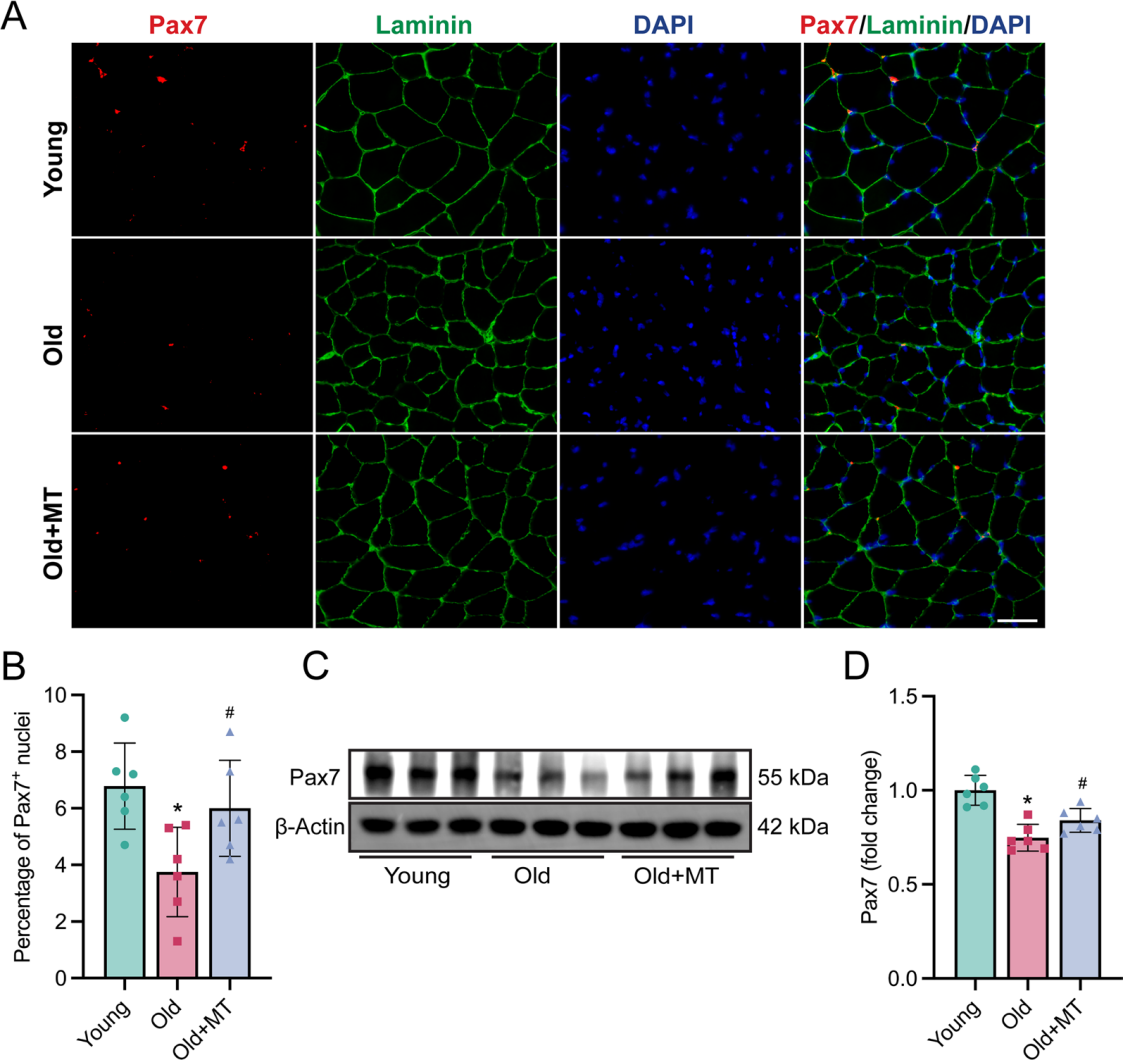
Melatonin restored satellite cell pool in old mice. **A** Representative images of immunofluorescence staining in gastrocnemius muscle, Pax7 (red), Laminin (green), and DAPI (blue) (Scale bar: 50 µm). **B** Analyses of number of Pax7 positive SCs. **C** Representative western blotting. **D** Densitometric analysis of Pax 7 in gastrocnemius muscle. The densities were normalized with those of β-Actin. Data represent mean ± S.D. of at least three independent experiments (n = 6 per/group). *p < 0.05 versus Young. ^#^p < 0.05 versus Old

The decline in SCs number contributes to impaired muscle atrophy in aging. To determine whether melatonin can restore the SCs pool in aged mice, we analyzed SCs numbers. Here, immunofluorescent staining for Pax7 revealed a significant reduction in Pax7-positive cells in aged mice compared to young controls, which was partially reversed by melatonin treatment (Fig. 2A, B). Western blot analyses showed that melatonin treatment increased the protein expression of Pax7 in muscle tissues (Fig. 2C, D). These findings suggest that melatonin restores the SCs pool in aged mice, which may contribute to combating sarcopenia.

### Melatonin reduced muscle fibrosis in old mice

**Fig. 3:**
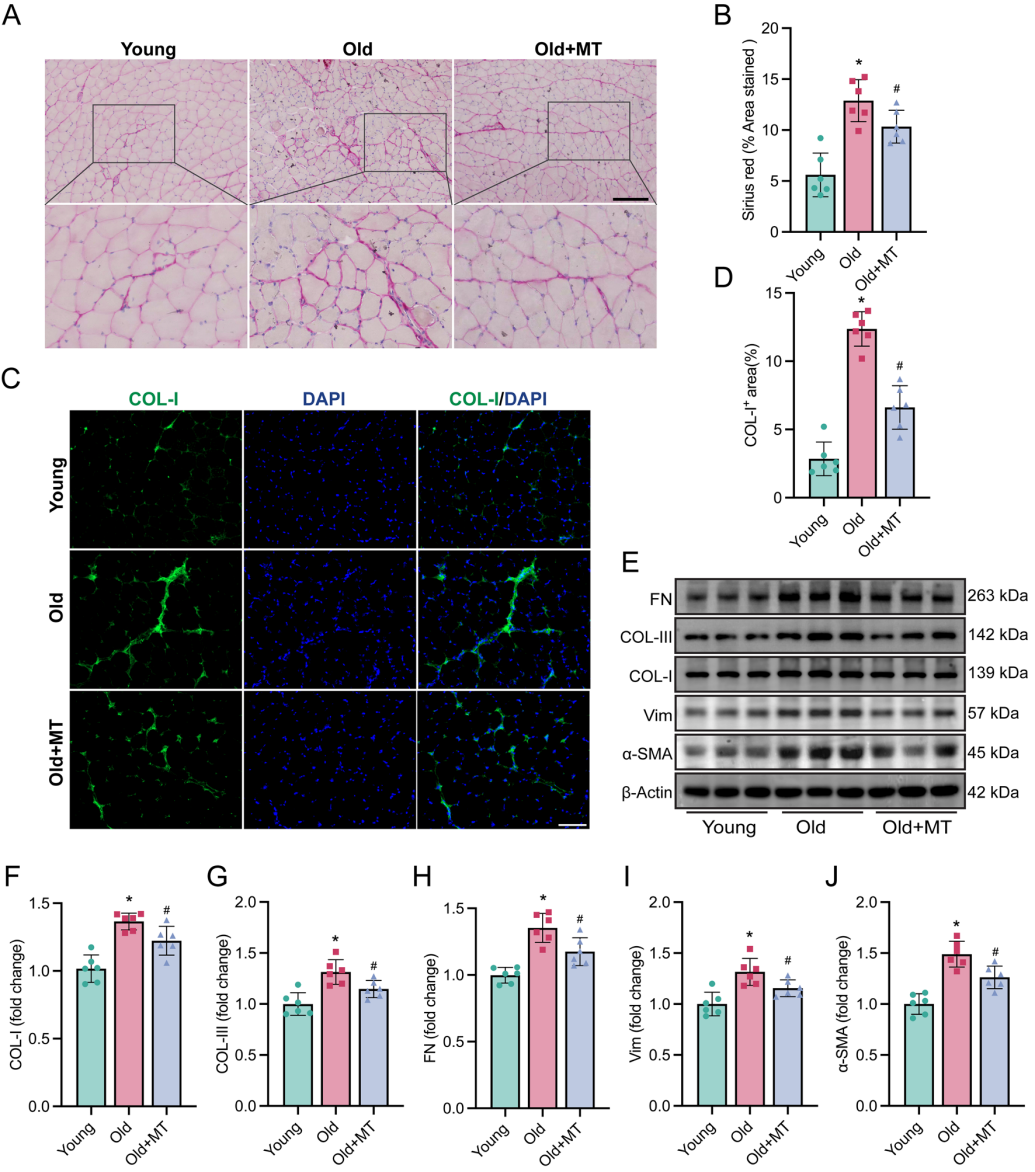
Melatonin reduced muscle fibrosis in old mice. **A**, **B** Representative images of sirius red staining of gastrocnemius muscle (Scale bar: 200 µm). **C**, **D** Representative images of immunofluorescence staining showing COL-I (green) + DAPI (blue) of gastrocnemius muscle slides (Scale bar: 100 µm). **E** Representative western blotting. **F**–**J** Densitometric analysis of FN, COL-I, COL-III, Vim, and a-SMA in gastrocnemius muscle. The densities were normalized with those of β-Actin. Data represent mean ± S.D. of at least three independent experiments (n = 6 per group). *p < 0.05 versus Young. ^#^p < 0.05 versus Old

Fibrosis of skeletal muscle is a hallmark of sarcopenia and characterized by excessive accumulation of extracellular matrix (ECM). We assessed whether melatonin could mitigate muscle fibrosis in aged mice. Here, Sirius Red staining showed increased collagen accumulation in the muscle tissue of aged mice, indicative of heightened fibrosis, which was attenuated by melatonin treatment (Fig. 3A, B). Immunofluorescence staining for collagen I (COL-I) further confirmed reduced fibrosis with melatonin (Fig. 3C, D). Western blot analyses revealed that melatonin decreased the expression of fibrogenic markers COL-I, collagen III (COL-III), α-smooth muscle actin (α-SMA), fibronectin (FN), and vimentin (Vim) in muscle tissues (Fig. 3E–J). These results demonstrate that melatonin effectively reduces muscle fibrosis in aged mice, which may contribute to improved muscle function.

### Melatonin restored myogenic potential of aging satellite cells in vitro

**Fig. 4:**
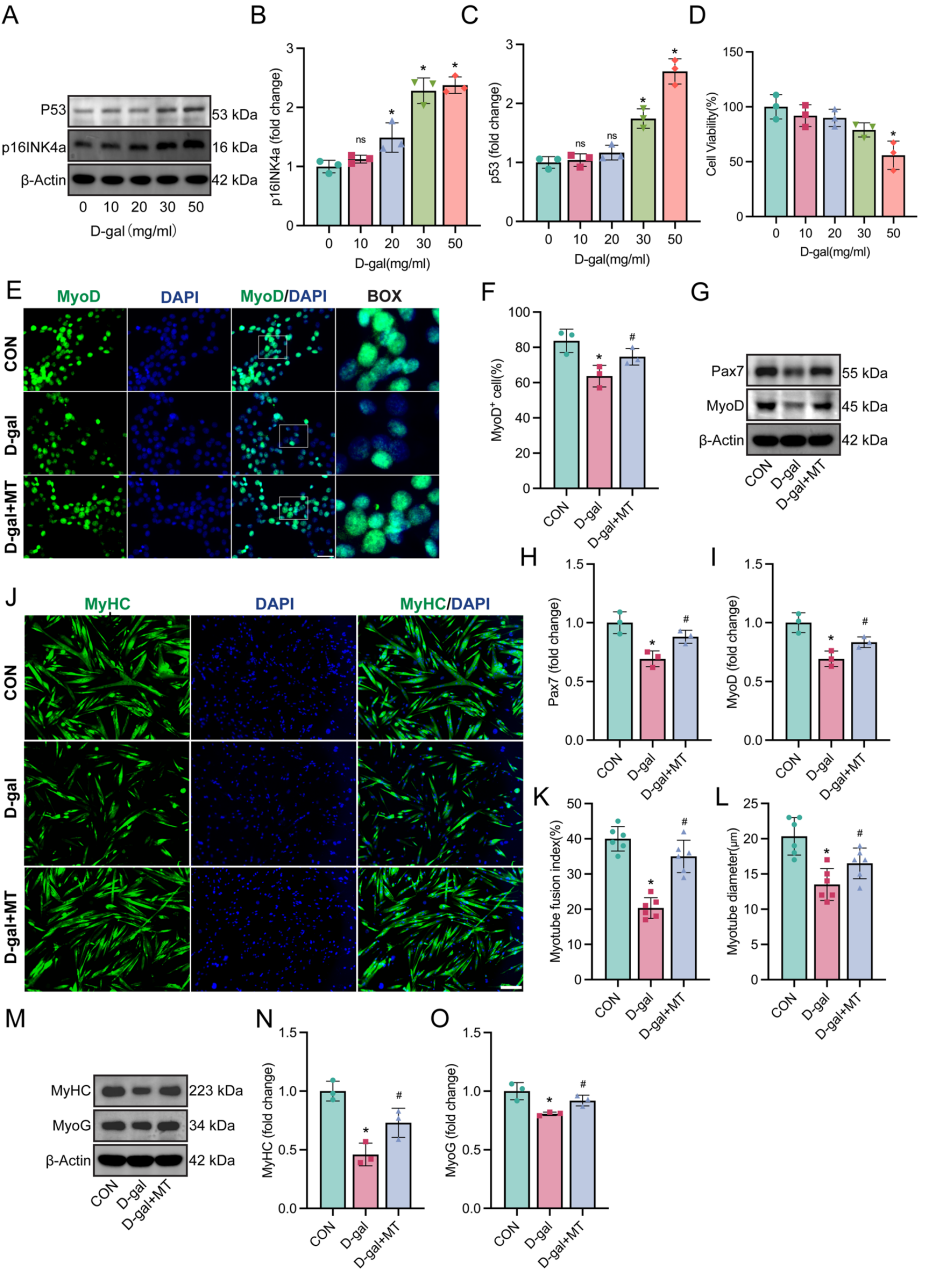
Melatonin restored myogenic potential of aging SCs. SCs incubate with different concentrations of d-gal for 24 h to obtain the most suitable concentration for aging induce. Then the SCs were categorized into three groups and culture in differentiate medium: Control group, Group treated with 30 mg/ml d-gal, and Group treated with both 30 mg/ml d-gal and 100 µM melatonin. **A**–**C** Representative western blotting and densitometric analysis of P53, p16INK4a in satellite cells after 24 h intervention with different concentrations of d-gal. **D** Cell viability of satellite cells after 24 h intervention with different concentrations of d-gal. **E** Representative image of immunofluorescence staining of MyoD (green) and nucleus DAPI (blue) in each group of cells after 24 h differentiation (Scale bar: 50 µm). **F** Analyses of the proportion of MyoD positive nuclei. **G**–**I** Representative western blotting and densitometric analysis of Pax7 and MyoD in each group of cells after 24 h differentiation. **J** Representative image of immunofluorescence staining of MyHC (green) and nucleus DAPI (blue) in each group of cells after 72 h differentiation (Scale bar: 20 µm). **K** Analyses of the fusion rate of differentiated SCs. The fusion index was defined as the number of nuclei in myotubes divided by the total number of nuclei present in the observed field. **L** Analyses of the diameter of myotubes in each group. **N**–**O** Representative western blotting and densitometric analysis of MyoG and MyHC proteins in each group of cells after 72 h differentiation. Data represent mean ± S.D. of at least three independent experiments (n = 3 per/group). **B**–**D** *p < 0.05 versus (0 mg/ml). (**F**, **H**, **I**, **K**, **L**, **M**–**O**) *p < 0.05 versus CON, ^#^p < 0.05 versus d-gal

Aging satellite cells exhibit diminished myogenic potential and increased fibrogenic conversion. To investigate whether melatonin can restore the myogenic potential of aging SCs, we established an in vitro aging model using primary SCs treated with d-gal, as outlined in previous studies (Wu et al. 2022; Azman and Zakaria 2019). As illustrated in Fig. 4A–D, d-gal treatment increased the expression of senescence markers P53 and p16^INK4a and reduced cell viability, confirming successful induction of cellular aging. For subsequent experiments, a concentration of 30 mg/ml d-gal was chosen to ensure aging induction without causing substantial harm to cell viability. The decision to use a 100 µM dose of melatonin was based on previous studies (Kim et al. 2011).

After 24 h of differentiation, by using MyoD to identify myogenic cells, we observed a decline in the MyoD-positive rate in SCs treated with d-gal compared to control group SCs (Fig. 4E–F). Western blot analyses also revealed decreased expressions of Pax7 and MyoD (Fig. 4G–I). However, melatonin effectively counteracted these reductions (Fig. 4E–I). After 72 h of differentiation, melatonin treatment enhanced myotube formation, increased fusion rates, and increased myotube diameters compared to aging SCs without melatonin (Fig. 4J–L). Western blot analysis confirmed that melatonin increased the expression of MyoG and MyHC in aging SCs (Fig. 4M–O). These results confirm that aging SCs exhibits a decline in the myogenic potential. Remarkably, a treatment with 100 µM melatonin partially mitigated these adverse changes.

### Melatonin inhibited fibrogenic differentiation in aging satellite cells

**Fig. 5:**
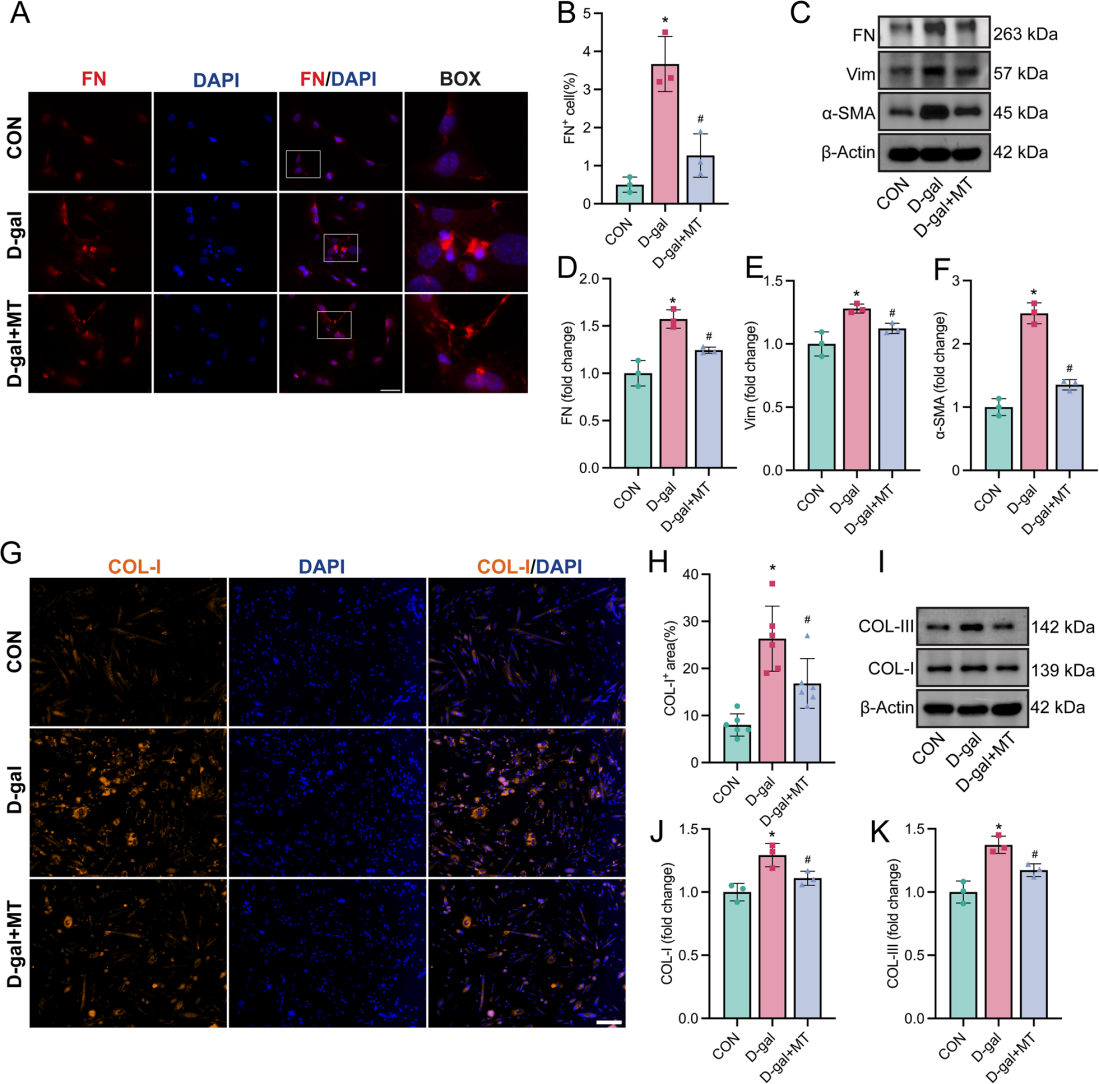
Melatonin inhibited fibrogenic differentiation in aging SCs. The SCs were categorized into three groups and culture in differentiate medium: Control group, Group treated with 30 mg/ml d-gal, and Group treated with both 30 mg/ml d-gal and 100 µM melatonin. **A** Representative image of immunofluorescence staining of FN (red) and nucleus DAPI (blue) in each group of cells after 24 h differentiation (Scale bar: 50 µm). **B** Analyses of the proportion of FN positive nuclei. **C**–**F** Representative western blotting and densitometric analysis of FN, Vim, and a-SMA in each group of cells after 24 h differentiation. **G** Representative image of immunofluorescence staining of COL-I (orange) and nucleus DAPI (blue) in each group of cells after 72 h differentiation (Scale bar: 20 µm). **H** Analysis of the COL-I positive area ratio in differentiated SCs. **I**–**K** Representative western blotting and densitometric analysis of COL-I and COL-III in each group of cells after 72 h differentiation. Data represent mean ± S.D. of at least three independent experiments (n = 3 per/group). *p < 0.05, **p < 0.01 versus CON, ^#^p < 0.05, ^##^p < 0.01 versus d-gal

Given that fibrogenic conversion of SCs contributes to muscle fibrosis in aging (Stearns-Reider et al. 2017; Zhou et al. 2019; Maltzahn et al. 2012), we assessed whether melatonin can inhibit fibrogenic differentiation of aging SCs in vitro. After 24 h of differentiation, d-gal treated SCs exhibited a conspicuous upsurge in the expression of fibronectin, a marker denoting fibroblastic cell morphology (Fig. 5A, B). This increase was further corroborated by Western blot assays, revealing augmented protein levels of fibronectin (Fig. 5C and D), along with another two fibrogenic markers, α-SMA (Fig. 5C and E), and vimentin (Vim) (Fig. 5C and F). Notably, these alterations were partially mitigated by treatment with 100 µM melatonin (Fig. 5A–F). After 72 h of differentiation, immunofluorescence highlighted elevated COL-I expression in d-gal-induced aging cells (Fig. 5G, H). Moreover, western blot analysis revealed that protein expressions of COL-I and COL-III were higher in the d-gal induction group compared to the control group (Fig. 5I–K). Melatonin treatment effectively mitigated these increases (Fig. G-K). These findings illustrate that aging SCs exhibit an increase in fibrogenic markers and ECM secretion, indicative of fibrogenic conversion. However, melatonin effectively counteracts this process.

### Melatonin suppressed TGF-β1 signaling both in vivo and in vitro

**Fig. 6:**
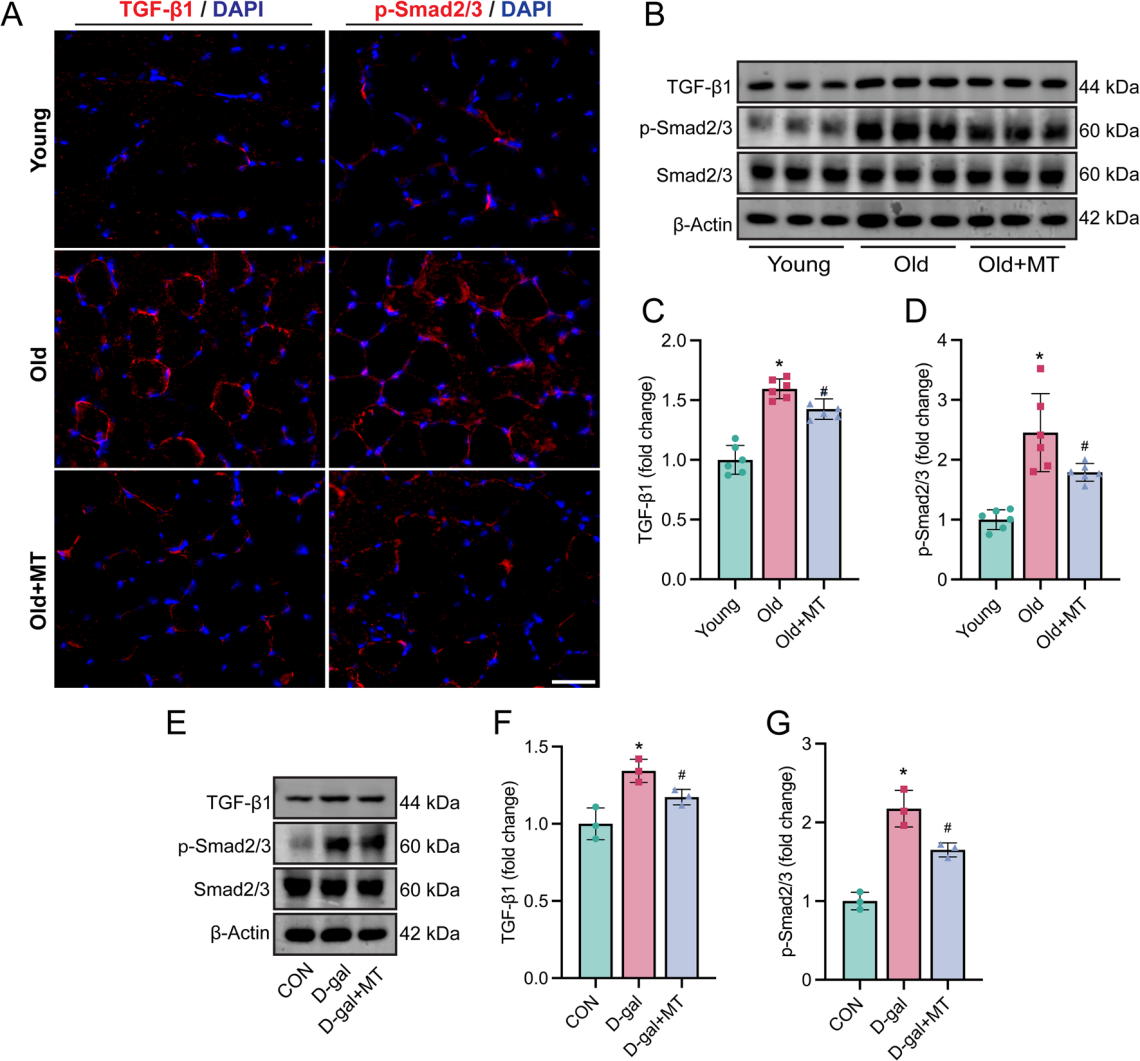
Melatonin suppressed TGF-β1/Smad2/3 Signaling in old Mice and d-gal induced aging SCs. **A** Representative image of immunofluorescence staining of TGF-β1/Smad2/3 (red) and nucleus DAPI (blue) of gastrocnemius muscle slides (Scale bar: 100 µm). **B**–**D** Representative western blotting and densitometric analysis of TGF-β1, Smad2/3, and p-Smad2/3 in gastrocnemius muscle of young mice, old mice, and melatonin-treated old mice. **E**–**G** SCs were differentiate 24 h in Control group, Group treated with 30 mg/ml d-gal, and Group treated with both 30 mg/ml d-gal and 100 µM melatonin. Representative western blotting and densitometric analysis of FN, Vim, and a-SMA in each group of cells. Data represent mean ± S.D. of at least three independent experiments. **C**, **D** *p < 0.05 versus Young. ^#^p < 0.05, versus Old, (n = 6 per/group). **F**, **G** *p < 0.05 versus CON, ^#^p < 0.05 versus d-gal, (n = 3 per/group)

TGF-β1, a fibrogenic master cytokine, and its downstream Smad2/3 signaling play an important role in the muscle fibrosis and fibrogenic conversion of SCs (Li et al. 2004; Pessina et al. 2015). To explore the mechanism by which melatonin affects SC fate, we examined the TGF-β1 signaling pathway. Within muscle tissues, immunohistochemistry and western blot analyses showed increased expression of TGF-β1 and phosphorylation of Smad2/3 in muscle tissues of aged mice, which were reduced by melatonin treatment (Fig. 6A–D). In aging SCs, d-gal treatment increased TGF-β1 expression and Smad2/3 phosphorylation, while melatonin effectively suppressed this pathway (Fig. 6E–G). These findings indicate that melatonin suppresses the TGF-β1/Smad2/3 signaling pathway in aging muscle and SCs, potentially underlying its protective effects against fibrogenic conversion and muscle fibrosis.

## Discussion

With global aging, sarcopenia has imposed a substantial burden on individuals and society. Our study demonstrates a significant decline in muscle mass and muscle strength in elderly mice, presenting of age-related sarcopenia. Furthermore, melatonin emerges as an effective intervention against age-related sarcopenia in elderly mice, as evidenced by its ability to enhance muscle quality and satellite cell pool while reducing muscle fibrosis. Mechanistically, melatonin reverses the decline in myogenic differentiation and myotube formation in aging SCs, while also inhibiting their fibrogenic conversion and ECM secretion. Both in vivo and in vitro, melatonin suppresses the pro-fibrogenic signaling pathway TGF-β1/Smad2/3. In essence, melatonin improves muscle quality by preventing the fibrogenic conversion of aging stem cells, offering promising prospects for the treatment of age-related sarcopenia.

As a neurohormone, melatonin has been recognized for its multiple regulatory effects on muscles (Salucci et al. 2021). Daily intraperitoneal injections 30 mg/kg of melatonin have been shown to improve muscle strength in malnourished mice, raising the overall glutathione content while reducing the oxidized/reduced glutathione ratio, optimizing the muscle redox state (Hibaoui et al. 2011). Additionally, melatonin prevents castration-induced skeletal muscle atrophy via the IGF-I axis (Öner et al. 2008). In human trials, melatonin mitigates skeletal muscle oxidative stress and prolongs physical performance (Stacchiotti et al. 2020). Despite these findings, limited studies have focused on melatonin’s role in combating age-related sarcopenia. Our study revealed that while melatonin intervention did not significantly alter the body weight of old mice, it effectively restored the muscle weight, thereby counteracting muscle wasting in aged mice. It also improved muscle cross-sectional area (CSA) and fiber diameter, most importantly enhancing grip strength in aged mice. These results provide strong evidence for the therapeutic potential of melatonin in the treatment of age-related sarcopenia.

The onset and progression of sarcopenia are closely tied to the number and function of SCs, which are essential for muscle regeneration and repair. Unfortunately, aging leads to a reduction of SCs pool, undermining the muscle's innate repair mechanisms and accelerating the development of sarcopenia (Verdijk et al. 2014; Dumont et al. 2021; Hong et al. 2021). In this study, Pax7, the most widely used and evolutionarily conserved marker for SCs (Allouh et al. 2007; Yin et al. 2013), was employed to label SCs. Our results revealed a reduction in Pax7-positive SCs in aged mice. However, melatonin effectively reversed this reduction. This finding aligns with previous research on muscle injury repair, where melatonin enhances Pax7 expression to promote recovery (Su et al. 2023), indicating its potential to restore the satellite cell (SC) pool in aged mice.

The ECM plays a critical role in muscle force transmission, maintenance, and repair (Gillies and Lieber 2011). However, excessive ECM deposition leads to muscle fibrosis, thereby influencing muscle flexibility, contraction, and relaxation. Sarcopenia in aged mice is often accompanied by increased activation of fibrotic pathways, including Wnt/β-catenin and TGF-β signaling, which promote excessive ECM deposition (Gillies and Lieber 2011; Alameddine and Morgan 2016). Existing research indicates that in muscles, the ECM is predominantly composed of COL-I and COL-III proteins (Kovanen 2002), mainly distributed within the endomysium, perimysium, and epimysium (Yanke and Chubinskaya 2015). In our study, melatonin reduced the deposition of these collagens and downregulated fibrogenic markers like α-SMA, fibronectin, and vimentin, effectively reversing muscle fibrosis in aged mice. While past research has underscored melatonin's anti-fibrotic effects in contexts like thioacetamide-induced liver fibrosis (Lebda et al. 2018), bleomycin-induced pulmonary fibrosis (Lan et al. 2022), and kidney fibrosis in db/db diabetic mice (Fan et al. 2020), our study is the first to demonstrate its efficacy in combating muscle fibrosis associated with aging.

To faithfully simulate in vivo conditions, we opted for primary SCs over the immortalized C2C12 cell line for our cell experiments. In this study, we isolated individual muscle fibers by digesting the muscle tissue and then cultured these fibers in an adherent manner. As a result, satellite cells detached from the muscle fibers, successfully achieving the isolation of these cells (Fig. S1A). This culture technique minimizes contamination from unrelated cells. Additionally, pre-coating uncoated tissue culture dishes helped further remove fibroblasts, yielding a purified population of SCs. This method avoids potential cell damage from FASC methods, which can focus only on specific SCs subpopulations, as well as damage from the Percoll density gradient purification method (Che et al. 2011; Matsuyoshi et al. 2018), while maintaining their inherent characteristics and gene expression profiles (Moyle and Zammit 2014; Rosenblatt et al. 1995; Conboy and Conboy 2010). Flow cytometry confirmed that over 96% of our isolated SCs expressed Pax7 (Fig. S1B-C), which is consistent with previous reports indicating that SC purity obtained using this technique typically ranges from 95 to 99% (Price et al. 2014; Sherwood et al. 2004; Maesner et al. 2016).

SCs fibrogenic conversion plays an important role in the development of age-related sarcopenia. Previous research has linked hyperphosphatemia in aging with reduced myogenic factors and increased fibrosis-related markers (Sosa et al. 2021). The Wnt signaling pathway has also been implicated in promoting fibrogenic conversion in aging SCs (Brack et al. 2007; Biressi et al. 2014). The fibrogenic conversion of SCs can also be attributed to their microenvironment; for instance, exposure to an aging extracellular matrix could suppress myogenesis while promoting fibrogenic conversion (Stearns-Reider et al. 2017; Zhou et al. 2019). Our findings indicate that d-gal-induced aging SCs undergo fibrogenic conversion, characterized by reduced myogenic potential and increased ECM secretion. Melatonin, however, protected against this conversion, providing strong evidence of its regulatory role in SC fate determination and its therapeutic potential in sarcopenia.

Although interstitial fibroblasts and fibro-adipogenic progenitors (FAPs) are the primary contributors to ECM deposition in aged muscles, it's vital to recognize the role of SCs in maintaining muscle homeostasis (Mann et al. 2011; Uezumi et al. 2011). Preventing SC fibrogenic conversion not only reduces ECM deposition but also helps preserve the SC pool, thus maintaining muscle regenerative capacity. This suggests that muscle atrophy and fibrosis may be driven by overlapping pathophysiological mechanisms. Therefore, rather than solely enhancing myogenic capacity, a deeper investigation into the triggers and modulators that promote the conversion of SCs from a myogenic to a fibrogenic fate could reveal novel therapeutic strategies for sarcopenia.

TGF-β signaling pathway is a canonical regulator involved in the fibrosis of tissues and organs (Ahmed et al. 2022; Lodyga and Hinz 2020). In injured muscle models, there is an upregulation of TGF-β expression in myogenic cells and directing myogenic cells toward a fibrogenic lineage (Li et al. 2004). Blocking TGF-β signaling with receptor antagonists can reverse this fibrogenic conversion (Pessina et al. 2015). Our study demonstrated that melatonin reversed the upregulation of TGF-β1 and Smad2/3 phosphorylation in aged mice and SCs. This suggests that melatonin modulates SC fate, at least partially, by inhibiting the TGF-β1/Smad2/3 pathway.

Despite our promising findings, this study has certain limitations that should be acknowledged. First, we did not perform muscle regeneration experiments using acute injury models such as cardiotoxin-induced injuries due to concerns about confounding inflammatory responses. Second, our in vitro use of d-gal-induced senescence in SCs does not fully replicate the complex aging processes occurring in naturally aged SCs. Attempts to use SCs from old mice were limited by their scarcity and reduced proliferative capacity, hindering comprehensive studies. Third, despite employing multiple purification techniques, it remains challenging to completely exclude the possibility of contamination from other cell types; thus, we cannot definitively confirm that all observed fibrogenic cells originated solely from SCs, although negative controls were included to address this issue. Additionally, melatonin may also impact myofibers and macrophages, not just SCs, and determining melatonin’s primary targets requires further investigation. Addressing these limitations in future work is crucial for understanding melatonin’s mechanisms of action and validating its potential therapeutic effects against sarcopenia.

## Experimental procedures

### Animal studies

All animals used in this study were purchased from the Southern Medical University Experimental Animal Centre (Guangzhou, China). Male C57BL/6J mice were divided into three groups: 1. Young group (3–5-month-old), 2. Old group (22–24-month-old mice), and 3. Melatonin group (22–24-month-old). For the Melatonin group, mice received daily intraperitoneal injections of 30 mg/kg (body weight) melatonin (Sigma-Aldrich, USA) for the last 3 months of their life. The Young and Old groups received daily intraperitoneal injections of saline solution. All mice were maintained in a specific pathogen-free environment under standard conditions of temperature (22 ± 2°), cycles of 12 h light/dark, and 54% relative humidity. Food and water were available ad libitum. One week before sacrifice, the mice's forelimb strength was measured by a grip test according to a standard protocol (TREAT-NMD SOP DMD_M.2.2.001). Body weight was recorded before sacrifice. Upon euthanasia, Gastrocnemius muscle (GA), Soleus Muscle (SOL), Tibialis Anterior muscle (TA) and Extensor Digitorum Longus (EDL) were collected, weighed, and processed for further analyses.

### Muscle cryosection

Muscles were embedded in Optimal Cutting Temperature (OCT) compound (Tissue-Tek®, USA), quickly frozen in liquid nitrogen-cooled isopentane for 2 min, and stored at −80 °C. Before sectioning on a cryostat (Leica CM1950, Germany), tissues were equilibrated for 30 min. Sections of 6 μm were obtained from the middle part of the muscle.

### Muscle fiber isolation

Single muscle fiber isolation was performed as described previously (Moyle and Zammit 2014). EDL from mice was digested in 0.2% type I collagenase (Sigma-Aldrich, USA) with gentle agitation for 2 h. Following digestion, muscles were shifted to a sterile biological safety cabinet. Using flame-sterilized wide-bore Pasteur pipettes, the muscle was transferred to dishes coated with 5% BSA and filled with Dulbecco's modified Eagle's medium (DMEM) (Gibco, USA) containing 4.5 g/l glucose. Muscle was triturated to release fibers, which were then transferred to fresh DMEM supplemented with 5% BSA for further analyses.

### Primary satellite cell isolation and culture

Primary satellite cell isolation was carried out as described previously (Rosenblatt et al. 1995). Isolated muscle fibers from the EDL muscle of 3–5-month-old mice were placed on a 10% matrix gel (Corning Technology, USA)-coated dish containing Dulbecco's Modified Eagle's Medium (DMEM) with GlutaMAX™ supplement (Gibco, USA). This medium was further supplemented with 20% fetal bovine serum (FBS) (Sigma-Aldrich, USA), 10% horse serum (Gibco, USA), 0.25% chick embryo extract (Absin, China), 5 ng/ml fibroblast growth factor 2 (MedChemExpress, China), and 1% penicillin/streptomycin (Gibco, USA). The muscle fibers were maintained in an environment of 5% CO_2_ at 37 °C.After 72 h, satellite cells (SCs) migrated from the attached muscle fibers to the surface of the matrix gel. At day 7, muscle fibers adhering to the dish were carefully removed using Pasteur pipettes. The remaining adherent SCs were digested with 0.25% trypsin (Gibco, USA). The resulting suspension was centrifuged, and the supernatant was discarded. The cells were then resuspended and transferred to an uncoated 100 mm culture dish. After 30 min of incubation, non-adherent cells were moved to a 10% matrix gel-coated dish. The purified cells were cryopreserved and stored at − 80 °C for subsequent use.

### Immunofluorescence staining of isolated muscle fibers and analyses

Muscle fibers were transferred to 1.5 ml centrifuge tubes under microscopic guidance. Fibers were fixed in 4% paraformaldehyde for 15 min, permeabilized with 0.5% Triton-PBS for 15 min, and blocked with 10% goat serum for 1 h. After removing the serum, fibers were incubated with Phalloidin-iFluor 594 Reagent (1:1000, Abcam, Cat# ab176757, UK) for 1 h at room temperature, then washed thrice in PBS for 5 min each. Fibers were mounted on slides using DAPI-containing medium and visualized using an upright fluorescence microscope (D2, Carl Zeiss, Germany). A total of 30 fibers per animal were measured to determine the average diameter using ImageJ.

### Immunofluorescence staining of muscle slides and cells and analyses

Simples were fixed in 4% paraformaldehyde for 15 min, washed thrice with PBS, permeabilized with 0.5% Triton-PBS solution for 15 min, and blocked with 10% goat serum for 1 h. After wash two time in PBS, incubated slides with primary antibodies (Paired Box 7 (Pax7) (1:20, DSHB, USA), Laminin (1:100, Abcam Cat# ab11575, UK), Collagen I (COL-I) (1:100, Abcam Cat# ab270993, UK), Fibronectin (FN) (1:100, Abmart Cat# PA3222M, China), Myosin Heavy Chain (MyHC) (1:20, DSHB Cat# MF 20, USA), TGF-β1 (1:200, Affinity Biosciences Cat# #AF1027, USA) and p-Smad2/3(Thr8) (1:200, Affinity Biosciences Cat# AF3367, USA)) overnight at 4 °C in a humidity chamber. The next day, slides were washed thrice with PBS, and incubated with the goat anti-rabbit IgG cross-adsorbed secondary antibody Alexa-Fluor® 594 conjugate (1:1000, ThermoFisher Scientific Cat# A11037, UK) or goat anti-mouse IgG cross-adsorbed secondary antibody Alexa-Fluor® 488 conjugate (1:1000, Abcam Cat# ab150117, UK) at room temperature for 1 h. After washing, slides were mounted using a DAPI-containing mounting medium. Images were acquired using an upright fluorescence microscope (D2, Carl Zeiss, Germany) or an inverted fluorescence microscope (IX73, Olympus, Japan), and analyzed using ImageJ software. Random fields of view were selected for each sample, and 500 DAPI-positive nuclei were counted. The percentages of Pax7-positive and FN-positive cells were calculated by dividing the number of Pax7-positive or FN-positive cells by the total number of DAPI-positive nuclei.

### Hematoxylin and eosin (H&E) staining and analyses

Slides rinse in PBS for 3 min to remove OCT. A subsequent rinse was conducted under a gentle stream of tap water for 1 min. Hematoxylin staining was applied for 30 s, followed by a 1-min tap water rinse. Nuclei were blued using a 20-s immersion in 1X PBS, followed by another 1-min tap water rinse. The slides were then sequentially immersed in 70% and 95% ethanol solutions, each for 30 s. Counterstaining was performed with alcoholic eosin for 30 s. A dehydration step involved transitioning the slides through two sequences of 95% ethanol and three of 100% ethanol, each for 15 s. Ultimately, slides were cleared with three cycles of xylene immersion, lasting 1 min each, and then cover slipped. Image was obtained by using a high-resolution slide scanner (Shengqiang Technology Co, Ltd, Shenzhen, China) and image were analyzed in image J. For GA muscle maximal cross-sectional areas (mm^2^), 4 animal per group were analyzed. For fiber cross sectional areas (μm^2^), 100 muscle fibers per section were analyzed, totaling 400 fibers per animal. All these parameters were analyzed using ImageJ software.

### Sirius red staining and analyses

Muscle slides were fixed with 4% paraformaldehyde for 15 min and stained with Sirius red solution (Solarbio, China) for 60 min. Following a brief wash in acidified water, sections were optionally counterstained with hematoxylin for 2 min. After dehydration through ethanol and xylene, the tissues were mounted under coverslips. Slides were subsequently scanned using a high-resolution scanner (Shengqiang Technology Co, Ltd, China). Using ImageJ software, we analyzed four non-consecutive areas per section to calculate the stained area (%). For each animal, 4 sections were analyzed.

### Aging SC model induced by d-galactose and treatment of melatonin

SCs were maintain in growth medium [Dulbecco’s modified Eagle’s medium (DMEM) with GlutaMAX™ supplement (Gibco, USA), supplemented with 20% fetal bovine serum (FBS) (Sigma, USA), 10% horse serum (Gibco, USA), 0.25% chick embryo extract (Absin, China), 5 ng/ml fibroblast growth factor 2 (MedChemExpress, China), and 1% penicillin/streptomycin (Gibco, USA)]. Aging SCs induced by d-gal as previously described (Wu et al. 2022). SCs incubate with different concentrations of d-gal for 24 h to obtain the most suitable concentration for aging induce.

In the following experiment, change the culture medium to low serum medium (DMEM with GlutaMAX™ supplement, supplemented with 2% horse serum (Gibco, USA) and 1% penicillin/streptomycin) for induce SCs differentiation. Concurrently, SCs were treated with 30 mg/ml d-gal, either with or without 100 µM melatonin treatment. DMSO was used as a vehicle control, and its final concentration was less than 0.1%.

### Cell viability assessment

SCs were plated in 96-well plates with a density of 5000 cells/well, achieving 50–60% confluency. Post reaching the desired confluency, the cells induced with a range concentration of d-gal (0 mg/ml, 10 mg/ml, 20 mg/ml, 30 mg/ml, 50 mg/ml) (Sigma-Aldrich, St. Louis, MO, USA) for 24 h. The viability of these cells was then determined using the cell counting kit 8 (Solarbio, Beijing, China). The data represent the results of three independent experiments, each with four replicates.

### Protein extraction and western blot analyses

For muscle tissue, fully lyse in a Tissue Lyser (Luka, Guangzhou, China). For cell samples, collect cells and fully lyse on ice in lysis buffer (20 mM Tris–HCl pH 7.5, 150 mM NaCl, 1 mM EDTA, 1 mM EGTA, 1% Triton X‐100, 0.1% sodium deoxycholate, 10 mM sodium pyrophosphate) with pH 7.9 containing protease inhibitor cocktail. Subsequently, the lysate was centrifuged at 13,000 rpm for 15 min at 4 °C. Collect the supernatant and evaluate protein concentration using the BCA assay kit (Beyotime, Shanghai, China). The remaining supernatant was mixed with loading buffer and denatured by boiling for 10 min. Protein samples were run onto sodium dodecyl sulfate‐polyacrylamide gels and transferred to a 0.22 μm polyvinylidene fluoride (PVDF) membrane (Immobilon-P, Millipore, Bedford, Massachusetts, USA). Membranes were blocked with 5% non‐fat dry milk in Tween Tris‐buffered saline (20 mM Tris–HCl, pH 7.5, 150 mM NaCl, 0.05% Tween‐20) for 1 h at room temperature. Then, the membrane was incubated overnight at 4 °C with primary antibodies (myogenin (MyoG) (1:100, DSHB Cat# AB_2146602, USA), myoblast determination protein 1 (MyoD) (1:100, DSHB Cat# D7F2, USA), Myosin Heavy Chain (MyHC) (1:100, DSHB Cat# MF 20, USA), Paired Box 7 (Pax7)(1:100, DSHB Cat# pax7, USA), P53(1:1000, Abcam Cat# ab131442, UK), p16INK4a(1:1000, Abcam Cat# ab189034, UK), Collagen I (1:1000, Abcam Cat# ab270993, UK), Collagen III(1:1000, Abcam Cat# ab184993, UK), α-smooth muscle actin(α-SMA) (1:1000, Abmart Cat# T51132S, China), Fibronectin(1:1000, Abmart Cat# PA3222M, China), Vimentin (1:500, Santa Cruz Cat# sc-66002, USA), TGF-β1 (1:1000, Affinity Biosciences Cat# #AF1027, USA), Smad2/3 (1:1000, Affinity Biosciences Cat# AF6367, USA) and p-Smad2/3 (Thr8) (1:1000, Affinity Biosciences Cat# AF3367, USA)). After being washed with Tween Tris‐buffered saline, the membranes were further incubated with horseradish peroxidase (HRP)-conjugated goat anti-rabbit or goat anti-mouse secondary antibody (1:10000, Thermo Fisher Scientific CAT# 31460, UK). Then, the blot was stripped and reprobed with a rabbit anti‐β-actin (1:2000, Affinity Biosciences Cat#AF7018, USA) antibody to normalize the protein levels. The immunoreactive bands were detected using Enhanced Chemiluminescence (ECL) system and the densitometry of protein bands was analyzed using ImageJ software.

### Statistical analyses

Statistical analyses were performed with Graph Pad Prism software version 8.2.1 (GraphPad Prism Software, La Jolla, CA, USA). For all animal experiments, n = 6, while cell experiments contained n = 3. Independently repeated experiments were performed at least in triplicate. Results are expressed as mean ± Standard Deviation (SD). All data were analyzed using a one-way analysis of variance (ANOVA) followed by Bonferroni's post-tests for multiple comparisons. P values of less than 0.05 were considered as statistical significance.

## Conclusion

Our study underscores melatonin's therapeutic efficacy in counteracting sarcopenia in the elderly mice and its inhibitory role in the fibrogenic conversion of aging SCs. These insights indicate the prospective clinical value of melatonin in managing age-related sarcopenia, necessitating further detailed exploration into its molecular mechanisms and pragmatic applications in human subjects.

## Supplementary Information

Supplementary Material 1.

## Data Availability

No datasets were generated or analysed during the current study.

## Abbreviations

SCs: Satellite cells
MT: Melatonin
d-gal: d-galactose
GA: Gastrocnemius muscle
SOL: Soleus muscle
TA: Tibialis anterior muscle
EDL: Extensor digitorum longus
OCT: Optimal Cutting Temperature
DMEM: Dulbecco’s modified Eagle’s medium
FBS: Fetal bovine serum
FN: Fibronectin
Vim: Vimentin
α-SMA: α-Smooth muscle actin
COL-I: Collagen I
COL-III: Collagen III
MyoG: Myogenin
MyoD: Myoblast determination protein 1
MyHC: Myosin Heavy Chain
PAX7: Paired Box 7
P53: Tumor protein P53
ECM: Extracellular matrix
TGFβ-1: Transforming growth factor beta-1
