# Supplementary material for: Melatonin ameliorates age-related sarcopenia by inhibiting fibrogenic conversion of satellite cell
Source: Mol Med. 2024 Nov 30;30:238. doi: 10.1186/s10020-024-00998-2 (PMC11607977; doi:10.1186/s10020-024-00998-2)
Supplement: Supplementary file 1 — Supplementary Material 1. [file 10020_2024_998_MOESM1_ESM.docx]

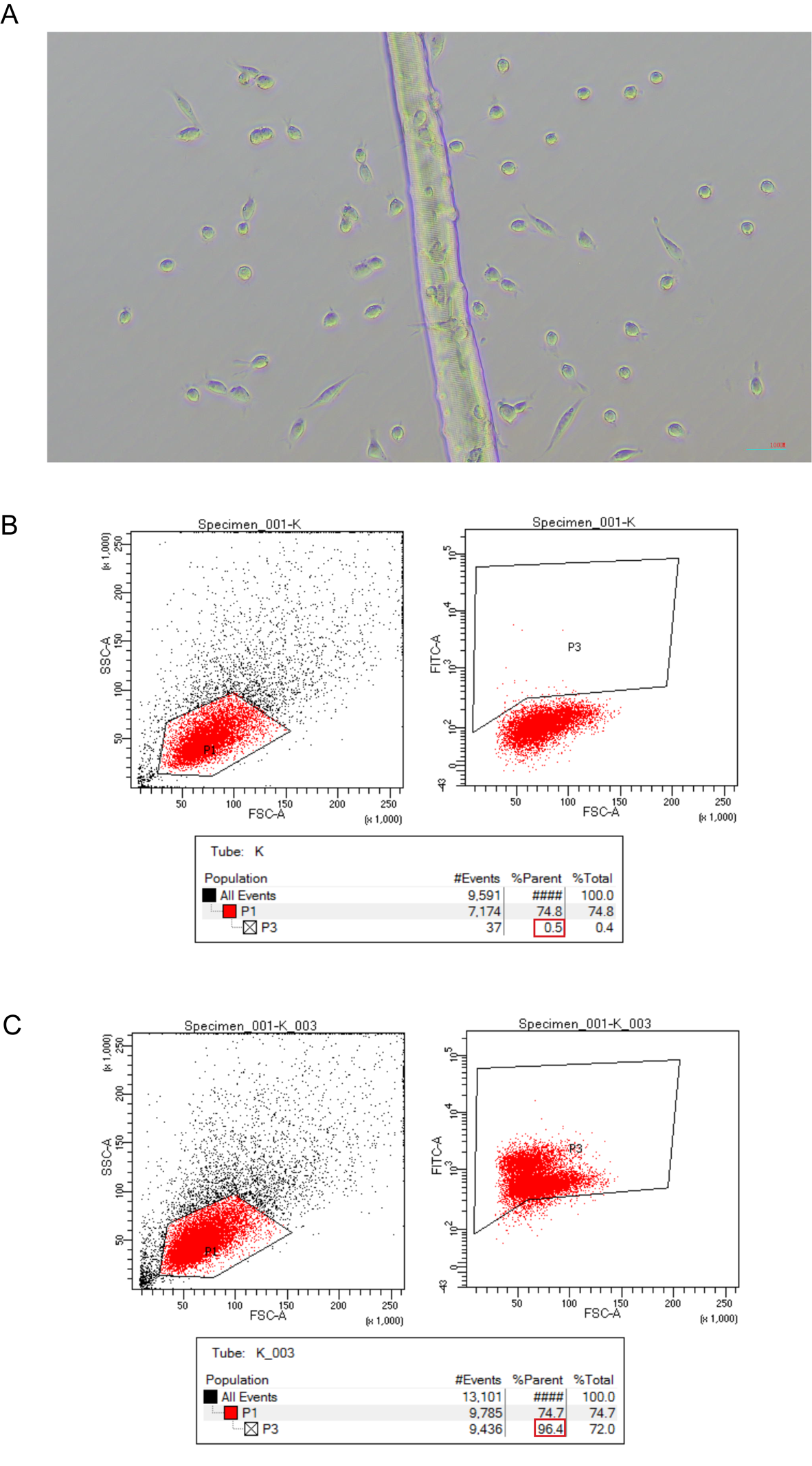


**Fig. S1.** Isolation of Satellite Cells. (A) Representative image of satellite cells migrating from muscle fibers. Cells were collected and immunostained with an anti-Pax7 antibody (1:50, DSHB, USA) at 4°C for 1 hour (cells in the negative control group were not subjected to immunostaining). This was followed by a 30-minute incubation with goat anti-mouse IgG cross-adsorbed secondary antibody Alexa-Fluor® 488 conjugate (1:1000, Abcam, Cambridge, UK). After washing, cells were analyzed using a BD FACS Verse flow cytometer. (B) represents the results of the flow cytometry assay for the negative control group, while (C) displays the results of the experimental group's flow cytometry assay.
